# Exergy and DFT-Based Thermodynamic Analysis of a Rankine–VCR Marine Waste Heat Recovery System

**DOI:** 10.3390/molecules31173065

**Published:** 2026-08-31

**Authors:** Arzu Keven, Enes Akçay, Hacer Gümüş

**Affiliations:** 1 Automotive Technology Program, Golcuk Vocational School, Kocaeli University, 41380 Kocaeli, Turkey; arzu.keven@kocaeli.edu.tr; 2 Department of Mechanical Engineering, Faculty of Engineering, Bayburt University, 69000 Bayburt, Turkey; enes_akcay.61@outlook.com

**Keywords:** Rankine-VCR, marine waste heat recovery, R448A, exergetic performance, quantum chemical analysis, DFT

## Abstract

This study investigates the performance of a diesel engine exhaust gas-driven Rankine Cycle-supported vapor compression refrigeration system using quantum chemical approaches beyond conventional thermodynamic analyses. The study focuses on R448A and its components, namely R32, R125, R134a, R1234yf, and R1234ze(E), evaluated at both the system level and the molecular level. At the system level, the Rankine–VCR system was analyzed using a Fortran-based macroscopic thermodynamic model, in which the thermophysical properties of the working fluids were obtained from the NIST Chemistry WebBook. At the molecular level, Density Functional Theory (DFT) calculations were performed to determine molecular structure parameters, including entropy, heat capacity, chemical hardness, and thermal enthalpy correction. The main objective is to investigate the possible relationships between these molecular descriptors and system-level performance indicators, while considering that mass flow rate is primarily governed by cycle thermodynamic properties. The results show that molecular stability and structural order are strongly associated with system performance. Among the R448A components, R32, with the highest chemical hardness (8.19 eV) and lowest molecular entropy (58.9 cal/mol K), exhibits the most favorable exergetic behavior and achieves the highest plant exergy efficiency of 44.92%. In contrast, R1234ze(E), chemically softer (η = 4.42 Ev) and higher in entropy, exhibits the lowest performance. Additionally, R32’s lower thermal enthalpy correction is associated with higher latent heat of vaporization under the selected operating conditions, reducing the required mass flow by approximately 60–70% compared to the other components. This study demonstrates that, in refrigerant selection, not only the global warming potential (GWP) but also quantum parameters such as chemical hardness, molecular entropy, heat capacity, and thermal enthalpy correction can serve as important complementary performance indicators when interpreted together with macroscopic thermodynamic and exergetic results.

## 1. Introduction

The increasing demand for energy and tightening environmental regulations necessitate the redesign of energy systems to enhance efficiency and sustainability. In this context, the recovery of waste heat in the maritime sector is of significant importance for reducing fuel consumption and mitigating greenhouse gas emissions [1]. Utilizing the low- and medium-temperature waste heat released from ship main engines and auxiliary systems has emerged as an effective solution to improve overall energy efficiency. Rankine Cycle systems enable power generation even at low temperatures due to the use of organic working fluids with low boiling points [2]. In addition, Vapor Compression Refrigeration (VCR) cycles are widely employed to meet the cooling demands of shipboard systems. Integrating Rankine and VCR cycles into Rankine–VCR-based systems provides a comprehensive solution, allowing simultaneous utilization of waste heat for both power generation and cooling purposes [3].

Performance evaluation of such integrated systems should not be limited to conventional energy analyses; exergy analysis based on the second law of thermodynamics plays a critical role in identifying actual losses and irreversibilities within the system [4]. Exergy-based assessments allow a detailed examination of component performance and the identification of potential improvements [5].

The influence of refrigerants on system performance is not restricted to macroscopic thermodynamic properties. At the molecular level, electronic structure characteristics, particularly chemical stability and interaction tendencies, are indirectly related to system irreversibilities. Quantum chemical calculations based on Density Functional Theory (DFT) enable the quantitative assessment of working fluids’ electronic behavior through HOMO–LUMO energy levels and derived global reactivity descriptors. This approach provides a multidisciplinary evaluation framework that spans from the molecular to the system scale [6].

In the present study, the performance of the R448A refrigerant in a Rankine–VCR-based marine waste heat recovery system was investigated using an integrated exergy- and molecular-level approach. Quantum chemical calculations were performed for the components of R448A, and the obtained molecular descriptors were correlated with system-level exergy analysis results, indicating statistical correlations between the working fluid’s electronic structure and its exergetic performance [7,8,9].

Although the literature shows a growing number of studies on the energy and exergy performance of Rankine–VCR-based systems, the integrated relationships between molecular-level electronic structures and system performance remain sparsely addressed [10]. The aim of this study is not to establish direct causal relationships between molecular descriptors and refrigeration performance. Instead, molecular descriptors obtained from quantum chemical calculations are used as complementary indicators to interpret differences among refrigerants within a multiscale thermodynamic framework.

## 2. Results and Discussion

**Figure 1:**
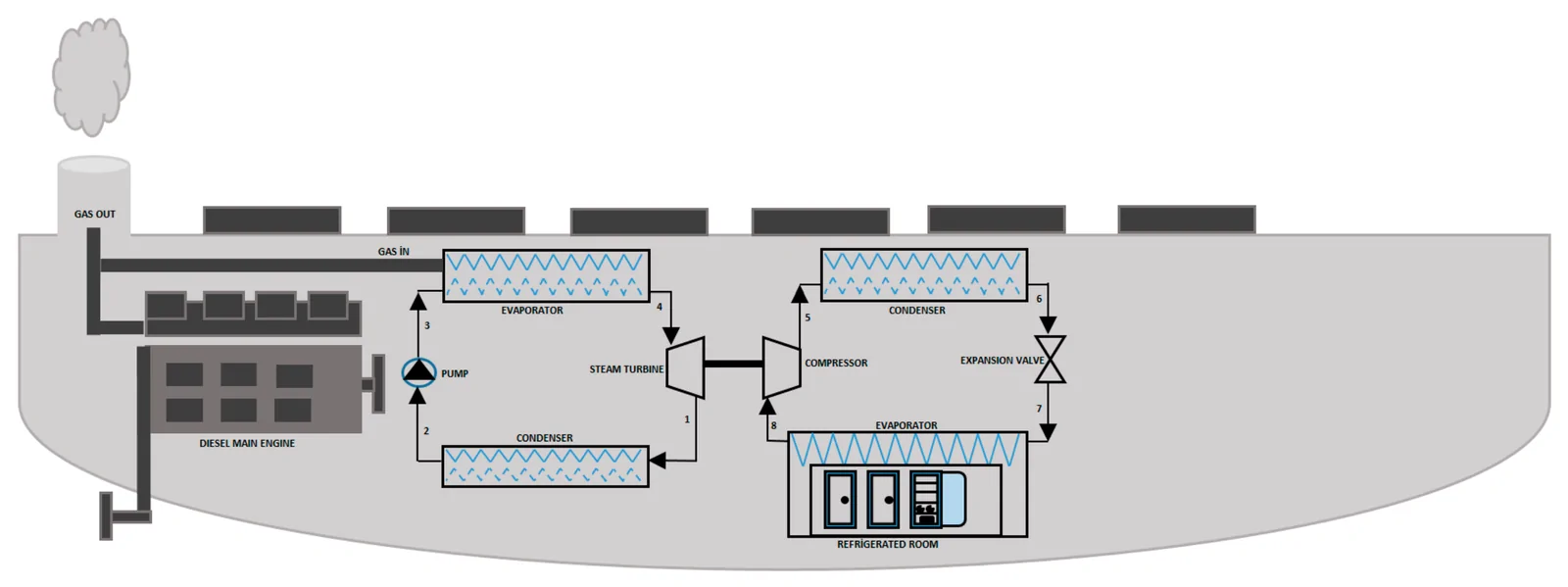
Schematic representation of the Rankine cycle–vapor compression refrigeration (VCR) system powered by exhaust gas from a marine diesel engine.

This study examines an integrated energy system designed to meet the ship’s air-conditioning and refrigerated-room demands by recovering waste heat from the exhaust gases discharged by the main diesel engine. A schematic representation of the system is presented in Figure 1.

The primary energy source of the system is the high-temperature exhaust gas discharged to the atmosphere through the ship’s funnel. In the Rankine cycle, the working fluid, namely water, is pressurized by the pump and then absorbs heat from the exhaust gas while passing through the evaporator/boiler, reaching the superheated steam state. The resulting high-pressure steam expands in the steam turbine and produces mechanical work.

In the present study, the shaft-coupled Rankine–VCR configuration is considered as a steady-state thermodynamic model to evaluate the waste-heat recovery potential of the proposed system. The mechanical power generated by the steam turbine is assumed to be transmitted to the refrigeration compressor through a shaft-coupling arrangement. However, in practical marine applications, fluctuations in engine exhaust conditions and variations in refrigeration demand would require an appropriate control strategy, such as a gearbox, clutch mechanism, bypass line, variable-speed compressor, or electrical buffer. Therefore, the proposed configuration should be interpreted as a thermodynamic feasibility model rather than a complete dynamic control design.

In the refrigeration block located on the right-hand side of the system, refrigerants such as R448A and its constituent components circulate as the working fluid of the VCR cycle. The compressor, driven by the recovered turbine power, compresses the refrigerant vapor. After rejecting heat in the condenser and undergoing pressure reduction across the expansion valve, the refrigerant absorbs heat in the refrigerated-room evaporator, thereby satisfying the ship’s cooling load.

### 2.1. System Parameters and Assumptions

To enable simulation of the thermodynamic model, the boundary conditions and component efficiencies were specified. Losses in rotating machinery, such as the turbine, pump, and compressor, were incorporated into the model via isentropic efficiency, while mechanical transmission losses between the Rankine and refrigeration cycles were represented by the shaft efficiency. The key design parameters, assumed efficiency values, and ambient conditions employed in the analyses are provided in Table 1.

The simulations were performed considering a constant cooling load of 20 kW. The compressor pressure ratio was varied between 3 and 7.5, while the exhaust gas inlet temperature was investigated in the range of 200–700 °C.

### 2.2. Thermodynamic Analysis Method and Governing Balance Equations

To determine the thermodynamic performance of the system, each component constituting the cycle was treated as an individual control volume. The analyses were conducted by applying the principles of mass conservation, energy conservation (First Law of Thermodynamics), entropy balance, and exergy balance (Second Law of Thermodynamics). The component-wise thermodynamic balance equations, along with the system performance metrics such as COP and exergy efficiency, are presented in Table 2. Silverfrost Fortran (FTN95) software was employed to solve the governing equation sets and to evaluate the thermodynamic properties of the working fluids.

### 2.3. Analysis of Molecular Structure

A zeotropic refrigerant mixture (R448A) is obtained by combining components belonging to the hydrofluorocarbon (HFC) and hydrofluoroolefin (HFO) classes in specific mass fractions. The mixture consists of difluoromethane (R32, 26%), pentafluoroethane (R125, 26%), 1,1,1,2-tetrafluoroethane (R134a, 21%), 2,3,3,3-tetrafluoropropene (R1234yf, 20%), and trans-1,3,3,3-tetrafluoropropene (R1234ze(E), 7%). The components forming this mixture exhibit different bonding types, saturated and unsaturated carbon–fluorine frameworks, and distinct electronic distribution characteristics. Therefore, investigating the structural and electronic properties of these molecules using quantum chemical methods is essential for understanding the molecular-level behavior of the mixture.

Since R448A is a multicomponent system, it is not possible to define a single crystal structure for the entire mixture. Accordingly, structural analyses were carried out based on the experimental crystal data and molecular geometries of the individual pure components archived in the Cambridge Structural Database (CSD/CCDC) [17].

Among the components of R448A, 1,1,1,2-tetrafluoroethane (R134a) has the chemical formula C_2_H_2_F_4_, and its crystal structure is recorded in the CCDC under the reference code 1288906. The R134a crystal belongs to the monoclinic crystal system with the space group P2_1_/c (No. 14). The unit cell parameters are a = 8.7569(4) Å, b = 9.3523(1) Å, c = 9.0784(4) Å, with cell angles α = 90°, β = 106.479(5)°, and γ = 90°. The experimental crystal structure data of R134a were reported by Brunelli et al. [18].

Similarly, the chemical formula of 2,3,3,3-tetrafluoropropene (R1234yf), another component of R448A, is C_3_H_2_F_4_, and its crystal structure is available in the Cambridge Structural Database. The R1234yf crystal crystallizes in the monoclinic system with the space group P2_1_/n (No. 14). The unit cell parameters are reported as a = 6.4246(14) Å, b = 8.1536(14) Å, and c = 8.913(3) Å, with cell angles α = 90°, β = 110.803(9)°, and γ = 90°. The corresponding crystal structure data were taken from the study published by Feller et al. [19].

**Figure 2:**
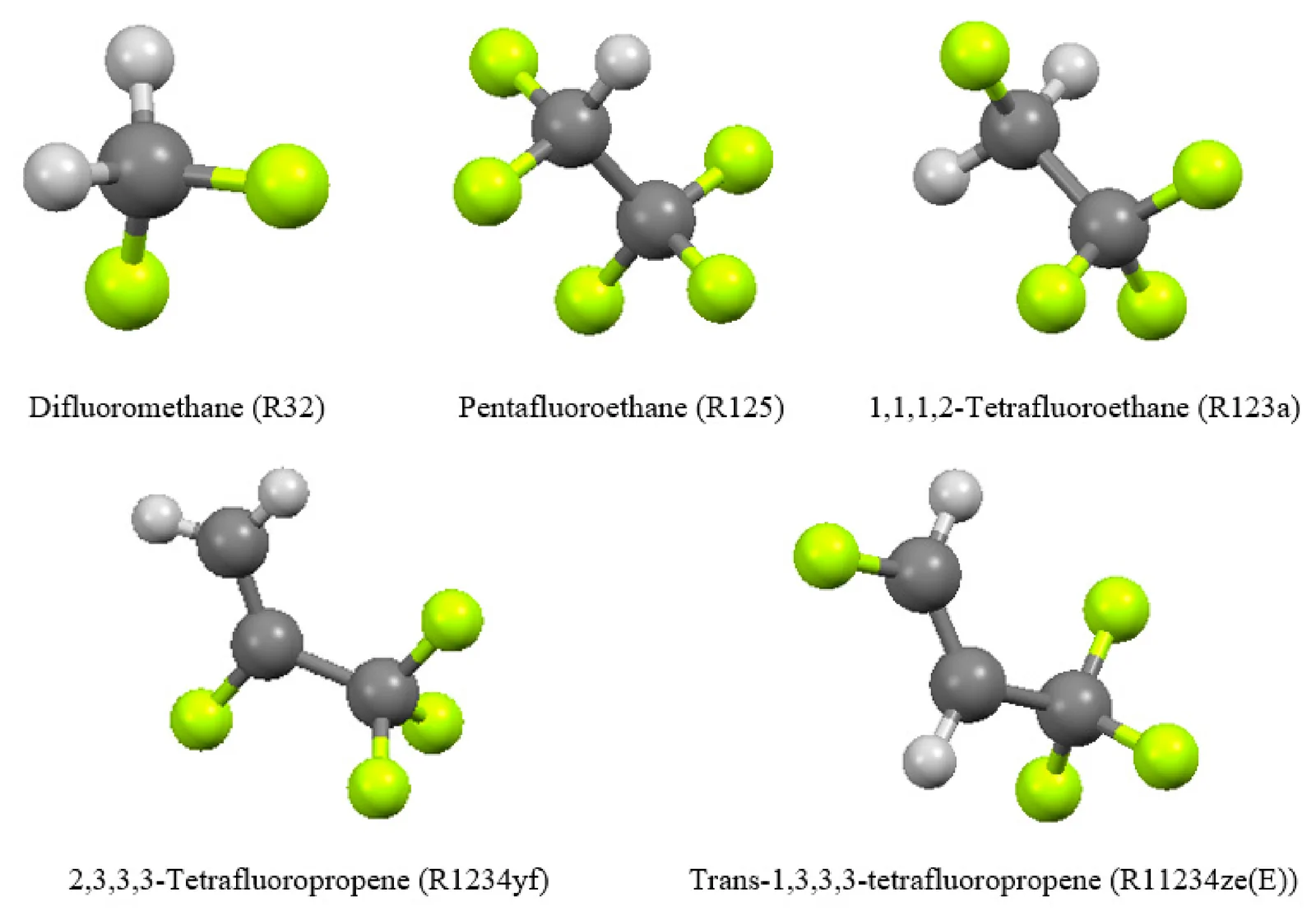
Molecular structures of the individual components of the R448A refrigerant, derived from X-ray crystallographic data reported in the Cambridge Structural Database (CCDC).

Finally, trans-1,3,3,3-tetrafluoropropene (R1234ze(E)), with the chemical formula C_3_H_2_F_4_, is also reported in the Cambridge Structural Database. The R1234ze(E) crystal belongs to the monoclinic crystal system with the space group P2_1_/m (No. 11). The unit cell parameters are a = 5.8460(4) Å, b = 6.5648(6) Å, and c = 10.7535(6) Å, with cell angles α = 90°, β = 90°, and γ = 90°. The experimental crystal structure data of R1234ze(E) were reported by Schwabedissen et al. [20]. In this study, the molecular structures were constructed based on the experimental crystal structure data reported in the CCDC and are illustrated in Figure 2.

The molecular structures of the components forming the R448A refrigerant (R32, R125, R134a, R1234yf, and R1234ze(E)) were constructed based on experimental crystal structure data reported in the Cambridge Structural Database (CCDC), as shown in Figure 2. Each component was subjected to geometry optimization in order to obtain stable configurations corresponding to minimum energy.

**Figure 3:**
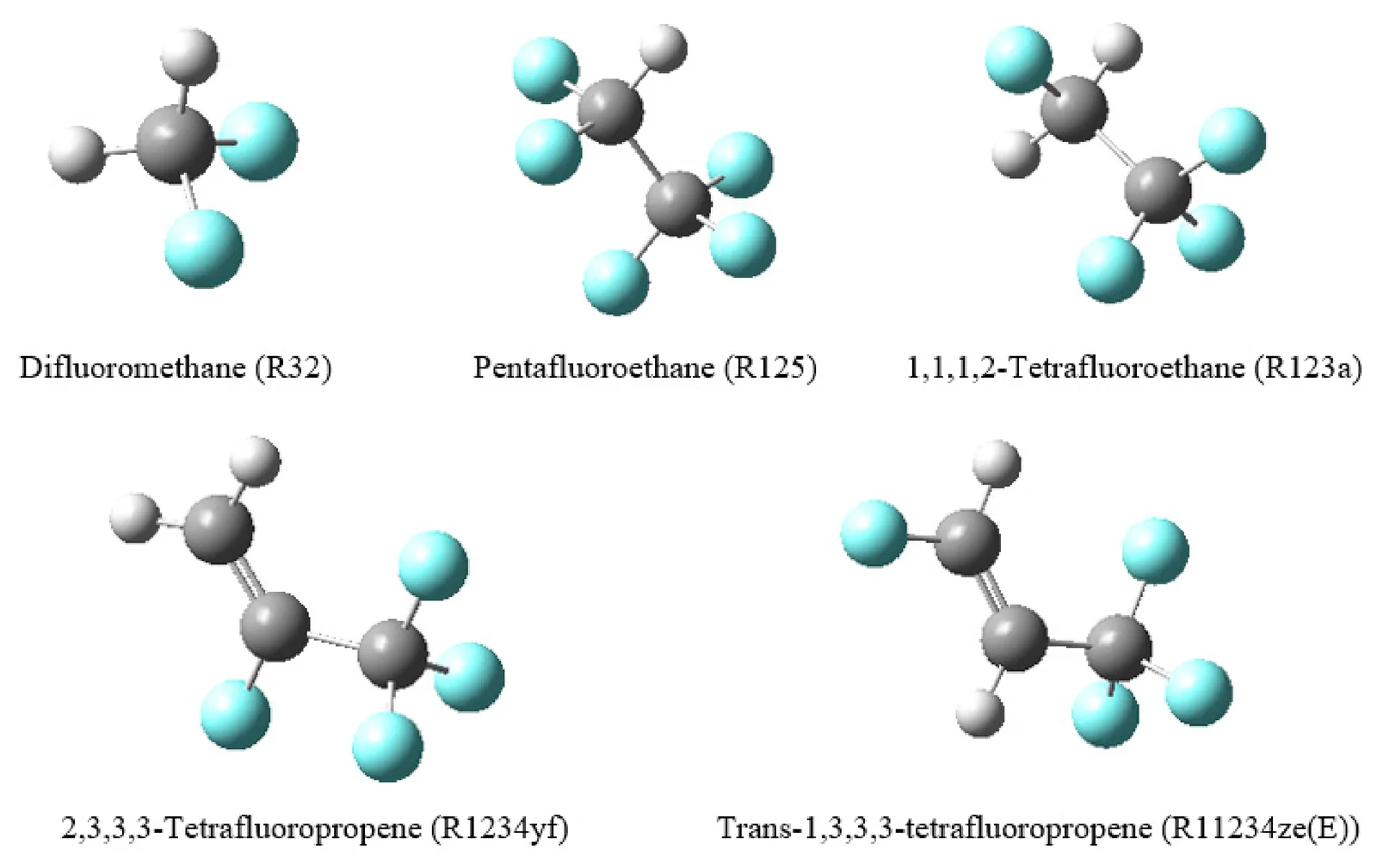
Optimized molecular structures of the components of the R448A refrigerant at the ωB97X-D/DGDZVP2 level of theory.

All molecules were modeled using the GaussView 6.1.1 program and optimized in the gas phase at the ground state within the framework of Density Functional Theory (DFT) using the ωB97X-D hybrid functional and the DGDZVP2 basis set as implemented in the GAUSSIAN 09 package. During the calculations, no external field or environmental effects were included, allowing the intrinsic molecular behavior of the isolated molecules to be examined independently of solid-state effects and crystal packing interactions. The optimized molecular geometries are presented in Figure 3.

In light of the optimized molecular geometries presented in Figure 3, the bond lengths and bond angles obtained from the DFT/(ωB97X-D/DGDZVP2) optimizations for all components constituting the R448A refrigerant (R32, R125, R134a, R1234yf, and R1234ze(E)) are found to be in good overall agreement with the corresponding experimental crystal structure data. This comparison demonstrates that the calculated geometric parameters are consistent with the experimental results.

Furthermore, the obtained results indicate that the symmetry and structural regularity exhibited by each component in terms of bond lengths and bond angles are in accordance with the experimental crystallographic data. The experimentally reported and calculated bond lengths and bond angles for all components of the R448A refrigerant (R32, R125, R134a, R1234yf, and R1234ze(E)) are presented in detail in Table 3.

The comparison of the experimental and theoretical geometric parameters for all components constituting the R448A refrigerant (R32, R125, R134a, R1234yf, and R1234ze(E)), as presented in Table 3, indicates an overall good agreement between the calculated bond lengths and bond angles and the experimental crystal structure data. The fact that the observed differences are largely limited to small deviations demonstrates that the employed DFT/(ωB97X-D/DGDZVP2) level of theory is capable of reliably describing the molecular geometries.

For R32, the deviation in C–F bond length was approximately 0.01 Å, while the F–C–F bond angle showed a difference of about 1.6°, demonstrating that the optimized geometry closely reproduces the experimental structure. In the case of R125, most C–F bond length deviations were within 0.005–0.015 Å, with bond angle differences generally below 1°. The high Pearson correlation coefficients obtained for both bond lengths (r ≈ 0.998) and bond angles (r ≈ 0.996) confirm the high accuracy of the calculated molecular geometry.

Similarly, for R134a, the differences between experimental and theoretical bond lengths were typically in the range of 0.02–0.03 Å, while bond angle deviations remained below 1°. The strong correlations observed for both bond lengths and angles further indicate that the molecular structure of R134a is reliably described by the applied DFT method.

For the unsaturated HFO refrigerants R1234yf and R1234ze(E), bond length deviations were generally within 0.01–0.03 Å, and most bond angle differences were smaller than 1°, with a few slightly larger deviations attributed to structural asymmetry and the presence of C=C bonds. Nevertheless, the Pearson correlation coefficients for both molecules remained very high (r ≥ 0.995), confirming the consistency between experimental and theoretical geometries.

### 2.4. Investigation of Thermodynamic Properties

In this study, the thermodynamic properties of selected components of the R448A refrigerant, namely R134a and R1234yf, were theoretically investigated using the Gaussian 09 program within the framework of Density Functional Theory (DFT) at the ωB97X-D/DGDZVP2 level of theory. These components were chosen as representative constituents of the R448A mixture in order to gain insight into the molecular-level thermodynamic behavior contributing to the overall performance of the refrigerant.

The calculations were carried out considering fundamental thermodynamic parameters, including thermal energy, heat capacity, entropy, rotational constants, and related properties. The contributions arising from translational, rotational, and vibrational molecular motions were evaluated separately, and total thermodynamic quantities such as internal energy, enthalpy, and entropy were subsequently determined. In addition, thermal corrections including zero-point energy (ZPE), energy corrections, enthalpy corrections, and Gibbs free energy corrections were taken into account. All calculated thermodynamic parameters are summarized in Table 4.

Table 4 summarizes the thermodynamic properties of all components constituting the R448A refrigerant mixture calculated at the DFT/ωB97X-D/DGDZVP2 level of theory. For all molecules, the translational and rotational energy contributions are identical, as expected for gas-phase calculations performed at the same temperature.

In contrast, the vibrational energy contribution increases systematically with molecular complexity, leading to higher total thermal energies for the unsaturated HFO components R1234yf and R1234ze(E), while the compact and symmetric R32 molecule exhibits the lowest values. A similar trend is observed for the heat capacity and entropy, where molecules with larger size and lower symmetry display higher vibrational contributions and total molar entropy.

**Figure 4:**
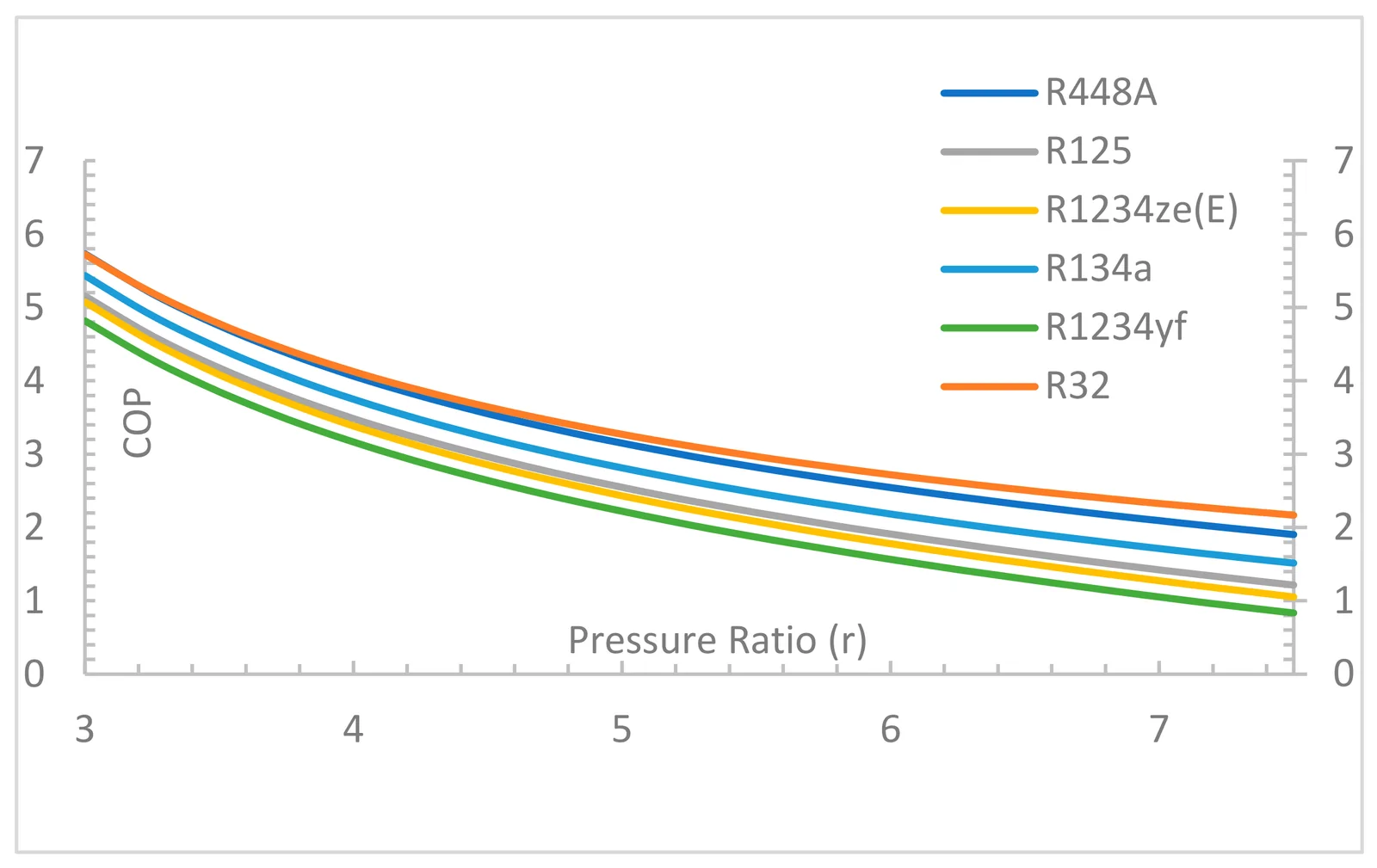
Effect of compressor pressure ratio (r) on the coefficient of performance (COP) of R448A and its constituent refrigerants.

Rotational constants and rotational temperatures further reflect the structural differences among the R448A components, with larger and more asymmetric molecules characterized by lower rotational constants. Overall, these results indicate an observed association between molecular complexity, vibrational contributions, and entropy within the investigated refrigerant components. These molecular descriptors are considered complementary indicators and should not be interpreted as direct causal determinants of system-level performance. In this context, the effect of compressor pressure ratio on the coefficient of performance (COP) for different refrigerants is discussed in Figure 4.

Figure 4 presents the variation in the system coefficient of performance (COP) with the compressor pressure ratio ranging from 3 to 7.5. Although the decrease in COP with increasing pressure ratio is a well-established thermodynamic behavior, the purpose of this analysis is to compare the relative performance response of R448A and its individual components under identical operating conditions. The observed differences among refrigerants provide a basis for evaluating the influence of molecular-level characteristics on system-level performance. As the pressure ratio increases, the COP decreases for all refrigerants due to the increase in specific compressor work under a constant cooling-load condition. Among the investigated refrigerants, R32 exhibits the highest COP over the entire pressure-ratio range, whereas R1234yf and R1234ze(E) show comparatively lower COP values. The R448A mixture provides a balanced performance compared with its individual constituent refrigerants.

**Figure 5:**
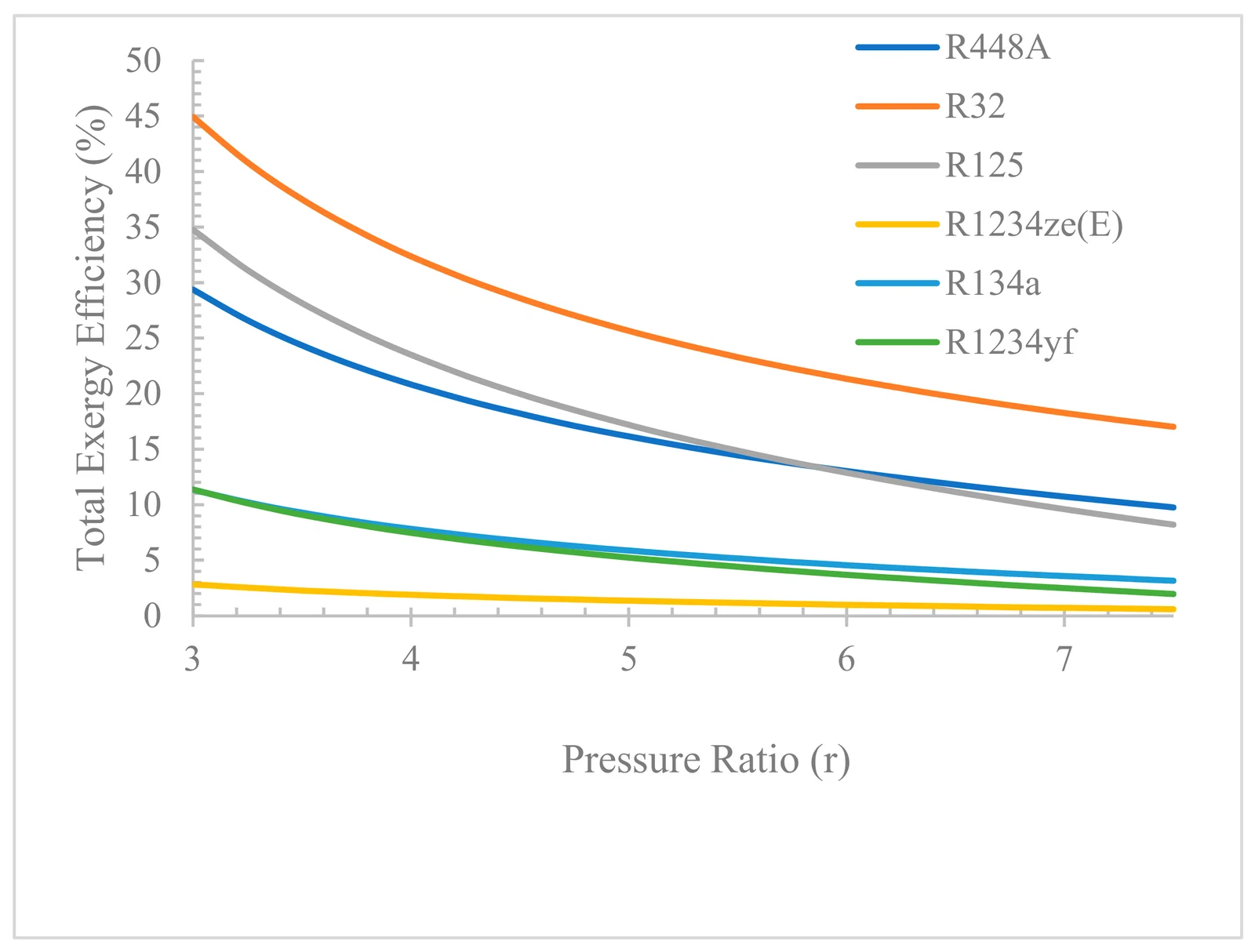
Effect of compressor pressure ratio (r) on the overall exergy efficiency of the R448A refrigerant and its components.

**Figure 6:**
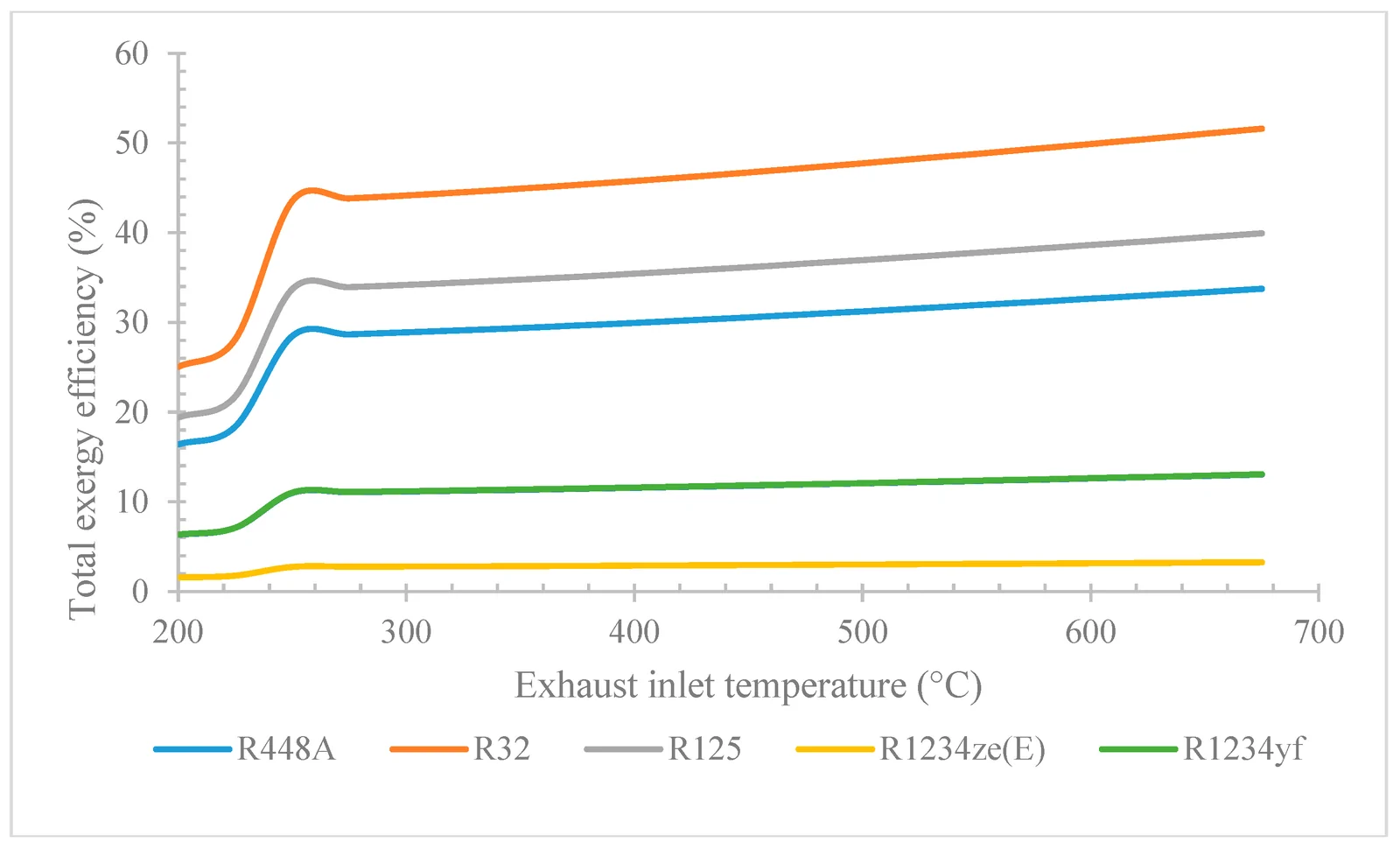
Effect of waste heat source (exhaust gas) inlet temperature on the overall plant exergy efficiency at a constant pressure ratio of r = 3.

Figure 5 shows the change in total exergy efficiency, representing the system’s Second Law performance, as a function of pressure ratio. It is observed that increasing the pressure ratio enhances irreversibilities (exergy destruction) in the system, leading to a significant drop in exergy efficiency. At low pressure ratios (r = 3), the exergy efficiency of R32 approaches 45%, whereas complex HFO refrigerants such as R1234yf and R1234ze(E) remain in the 5–12% range. The high efficiency of R32 is attributed to its low entropy generation and high heat transfer capacity. Although efficiencies of all refrigerants converge as the pressure ratio increases, R32 maintains its superior performance across the entire operating range. The subsequent figure (Figure 6) investigates the influence of the waste heat source temperature on the exergy efficiency of the R448A system.

Figure 6 illustrates the effect of exhaust gas inlet temperature, ranging from 200 to 700 °C, on the overall exergy efficiency of the Rankine–VCR system at a constant compressor pressure ratio of r = 3. As the exhaust gas temperature increases, the availability of the heat supplied to the Rankine cycle boiler increases, resulting in a general increasing trend in total exergy efficiency. However, the variation is not strictly linear, particularly in the lower temperature range, where changes in heat-source availability and cycle operating conditions affect the rate of increase. The relative performance ranking of the refrigerants remains almost unchanged over the investigated exhaust gas temperature range. R32 consistently exhibits the highest exergy efficiency, whereas R1234ze(E) shows the lowest values. This indicates that, under the selected operating conditions, both the refrigerant thermophysical properties and the waste-heat source temperature influence the overall exergy performance of the system.

**Figure 7:**
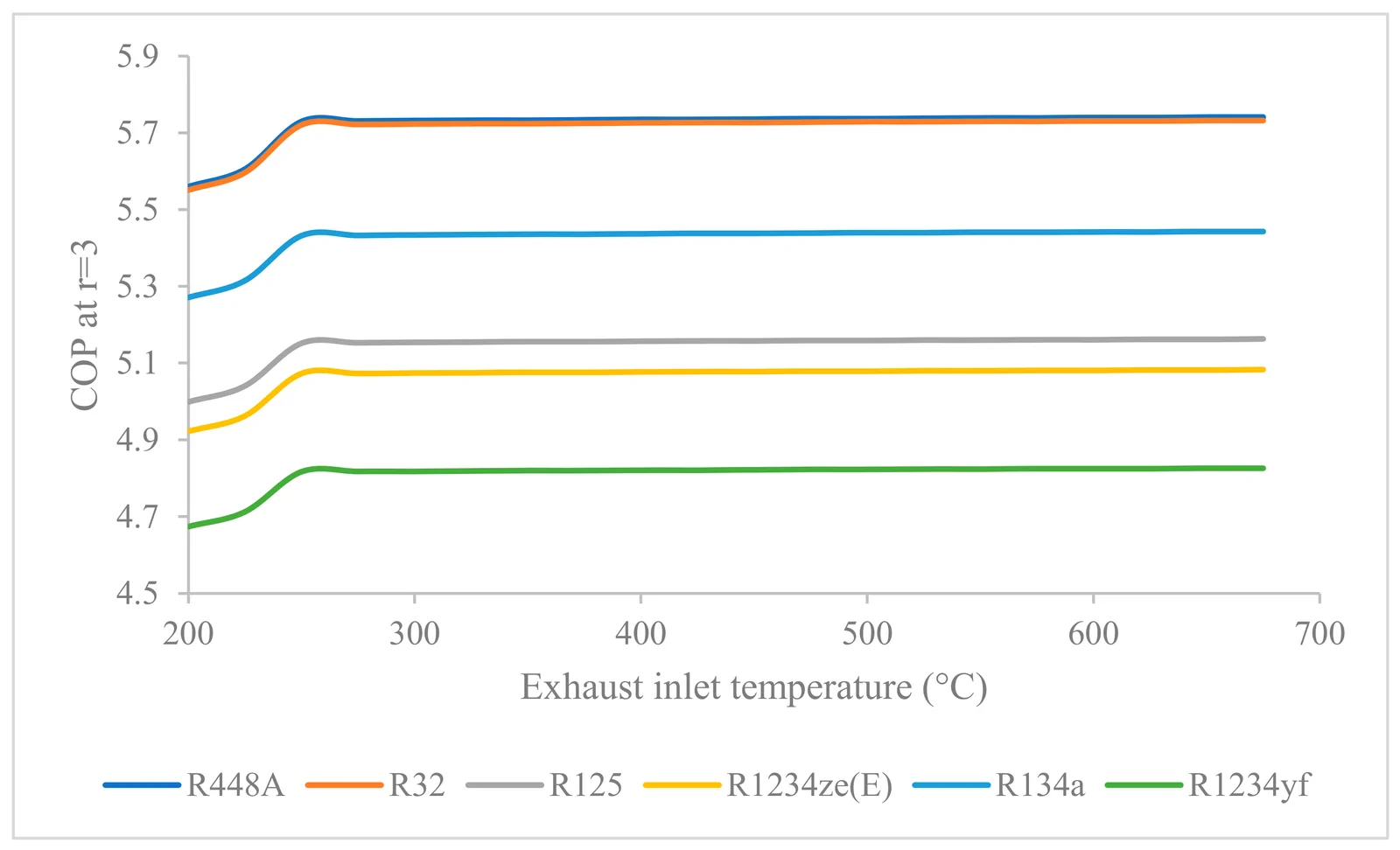
Effect of exhaust gas temperature on the coefficient of performance (COP) of R448A and its constituent refrigerants at r = 3.

Figure 7 shows the variation in the coefficient of performance (COP) with exhaust gas inlet temperature at a constant compressor pressure ratio of r = 3. The results indicate that the exhaust gas temperature has only a limited effect on the COP of the VCR subsystem. This is because the COP is mainly governed by the evaporator and condenser conditions of the refrigeration cycle rather than by the temperature level of the external waste-heat source. As long as the Rankine cycle provides sufficient mechanical power to drive the compressor, the refrigeration cycle operates according to its own thermodynamic conditions. Among the investigated refrigerants, R32 and R448A maintain relatively higher COP values, whereas R1234yf and R1234ze(E) exhibit comparatively lower performance. Therefore, refrigerant selection remains a key factor for maintaining stable cooling performance under varying exhaust gas temperatures.

**Figure 8:**
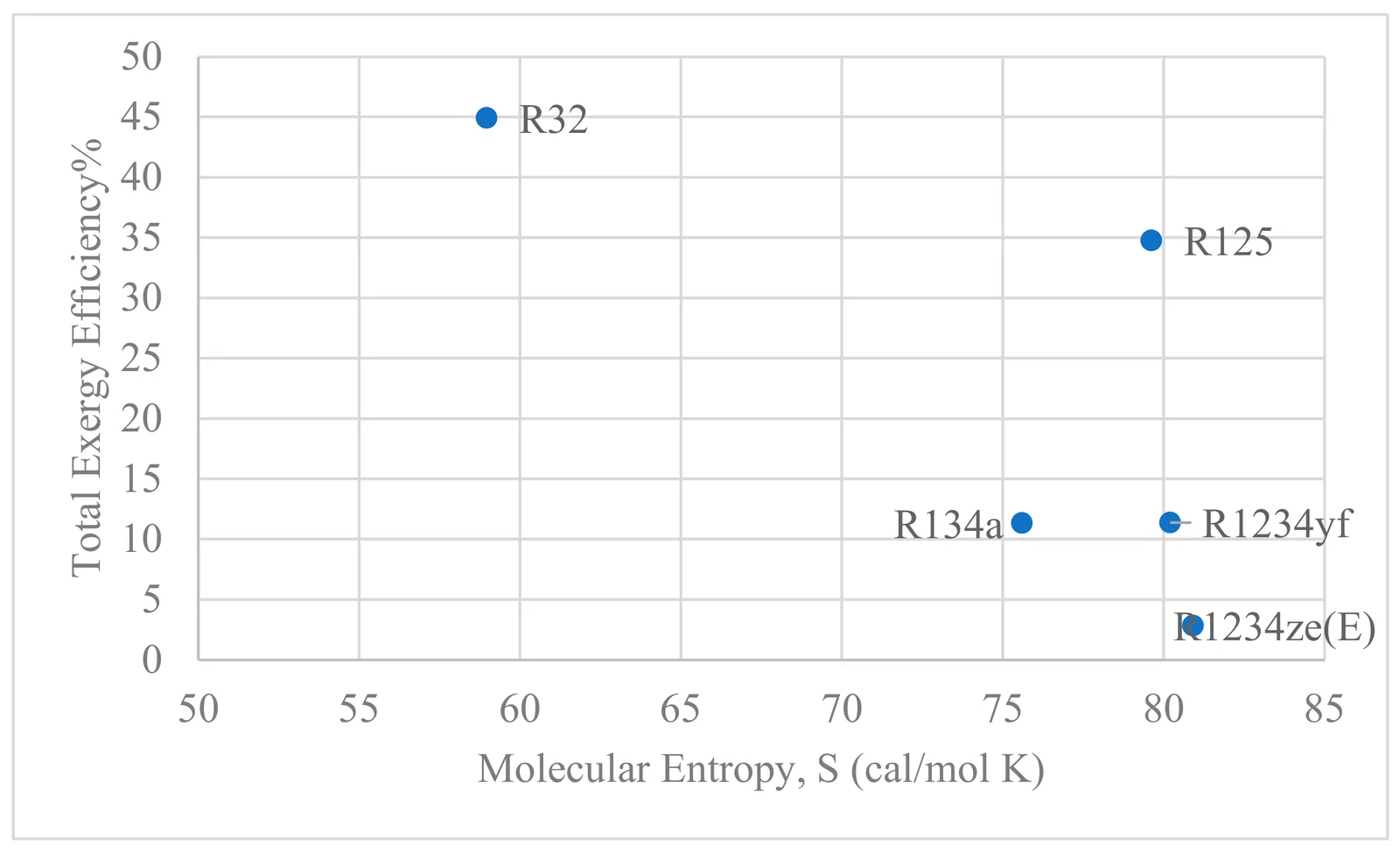
Relationship between molecular entropy and plant exergy efficiency for the R448A refrigerant and its constituent components.

Figure 8 presents the relationship between the DFT-derived standard molecular entropy values of the investigated refrigerants and the total exergy efficiency of the Rankine–VCR system. An inverse trend is observed within the limited set of refrigerants considered, with R32 exhibiting the lowest molecular entropy and the highest calculated exergy efficiency. However, this observed trend should be interpreted only as a descriptive association between a molecular-level descriptor and a system-level performance parameter. The molecular entropy values were obtained from isolated gas-phase DFT calculations, whereas the exergy efficiency was determined from the macroscopic thermodynamic cycle analysis. Therefore, the observed correlation does not establish that molecular entropy directly causes or predicts the system-level exergy efficiency. Differences in molecular entropy reflect variations in molecular size, symmetry, and vibrational and rotational contributions, while the calculated exergy efficiency is governed by the thermophysical properties and operating conditions of the complete thermodynamic cycle. Accordingly, molecular entropy is considered a complementary descriptor that may assist in interpreting the observed differences among the investigated refrigerants, rather than an independent performance criterion.

**Figure 9:**
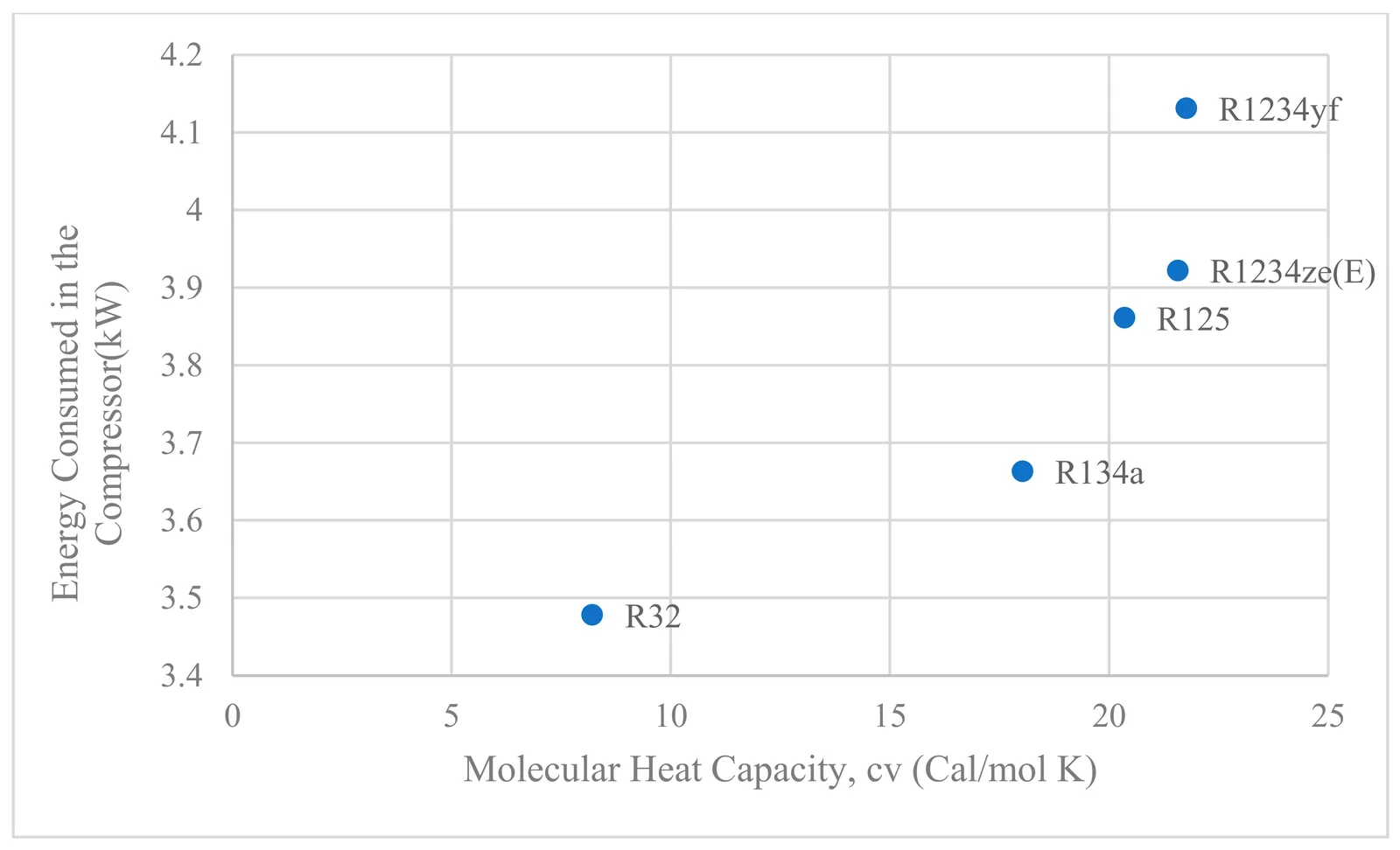
Relationship between molecular heat capacity (Cv) and mechanical work in the compressor.

Figure 9 presents the relationship between the DFT-derived molecular heat capacity (Cv) and the compressor work requirement of the investigated refrigerants. Within the limited set of refrigerants and the selected operating conditions, an apparent association is observed between the calculated molecular heat capacity and the compressor work. However, this association should be interpreted descriptively rather than as evidence of a direct causal relationship. From the system-level thermodynamic perspective, compressor work is determined by the refrigerant mass flow rate and the enthalpy rise across the compressor, expressed as ${\dot{W}}_{Comp}={\dot{m}}_{ref}{(h}_{5}-h_{8})$. The DFT-derived Cv values represent molecular-level thermal properties obtained under isolated gas-phase conditions and do not directly determine the compressor work of the refrigeration cycle. Therefore, the observed trend provides complementary molecular-level information for interpreting the differences among the refrigerants, while the actual compressor energy requirement remains governed by the macroscopic thermodynamic properties and operating conditions of the cycle.

**Figure 10:**
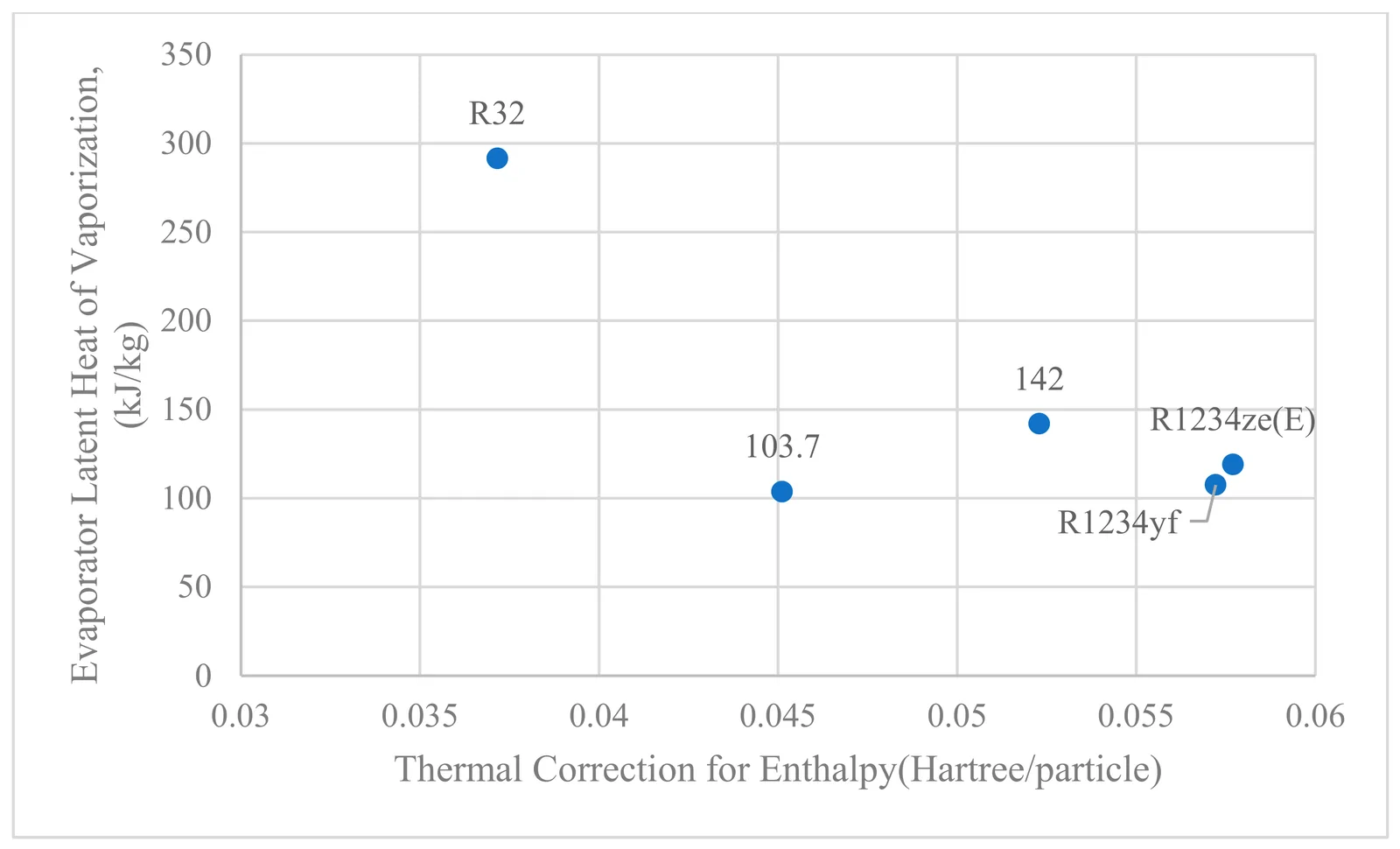
Thermal enthalpy correction versus latent heat of vaporization for R448A refrigerant components.

Figure 10 presents a qualitative comparison between the DFT-derived thermal enthalpy correction and the latent heat of vaporization of the investigated refrigerant components. The latent heat of vaporization is a bulk thermodynamic property determined by the thermophysical behavior of the refrigerant and intermolecular interactions associated with the liquid–vapor phase change. In contrast, the thermal correction to enthalpy obtained from DFT calculations represents molecular contributions under isolated gas-phase conditions. Therefore, no direct thermodynamic relationship between these two quantities is assumed in the present study. The comparison is presented only as a descriptive comparison to examine the apparent ordering of the two quantities within the limited set of refrigerants investigated. The observed ordering, particularly for R32, should therefore be regarded as a descriptive association rather than evidence of a causal or predictive relationship. The latent heat values used in the comparison were obtained from the macroscopic thermodynamic-property calculations, whereas the DFT thermal corrections were independently calculated from the molecular models.

**Figure 11:**
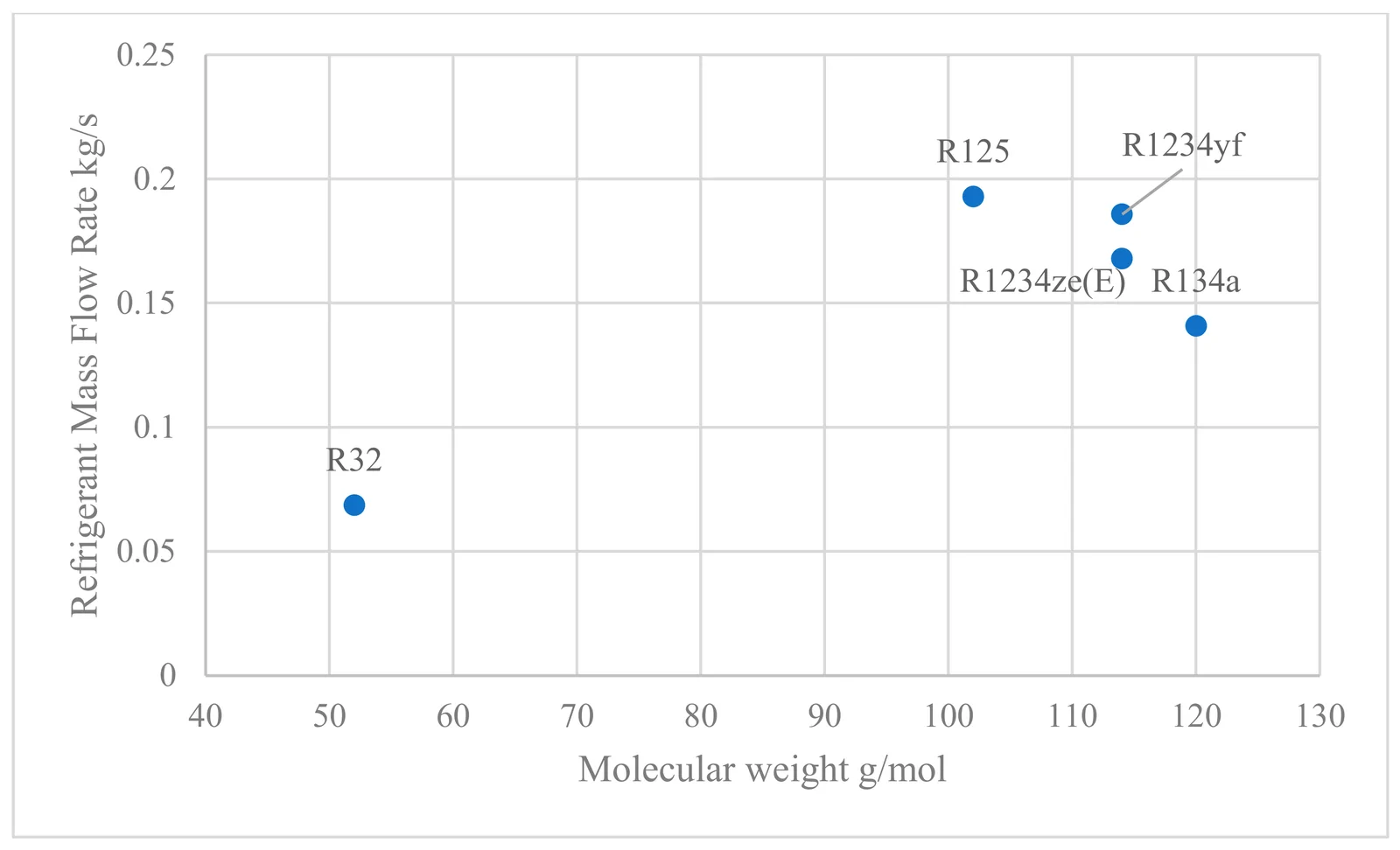
Effect of molecular weight on required mass flow rate for R448A refrigerant components.

Figure 11 presents the relationship between molecular weight and the required refrigerant mass flow rate for a constant cooling load of 20 kW. From the system-level thermodynamic perspective, the refrigerant mass flow rate is primarily determined by the specific cooling effect in the evaporator, expressed as ${\dot{m}}_{ref}={\dot{Q}}_{L}{(h}_{8}-h_{7})$. Therefore, refrigerants with a higher evaporator enthalpy difference require a lower mass flow rate to provide the same cooling capacity.

The observed trend indicates that molecular weight may show an apparent correlation with mass flow rate; however, the mass flow rate is fundamentally determined by the enthalpy difference across the evaporator. Lighter and structurally simpler refrigerants, such as R32, exhibit lower required mass flow rates under the selected operating conditions, mainly due to their favorable thermodynamic properties and higher specific cooling effect. In contrast, heavier components such as R125 require higher circulation rates to achieve the same cooling load. These results suggest that the molecular structure and thermophysical properties of the refrigerant jointly influence the required mass flow rate in Rankine–VCR refrigeration applications.

**Figure 12:**
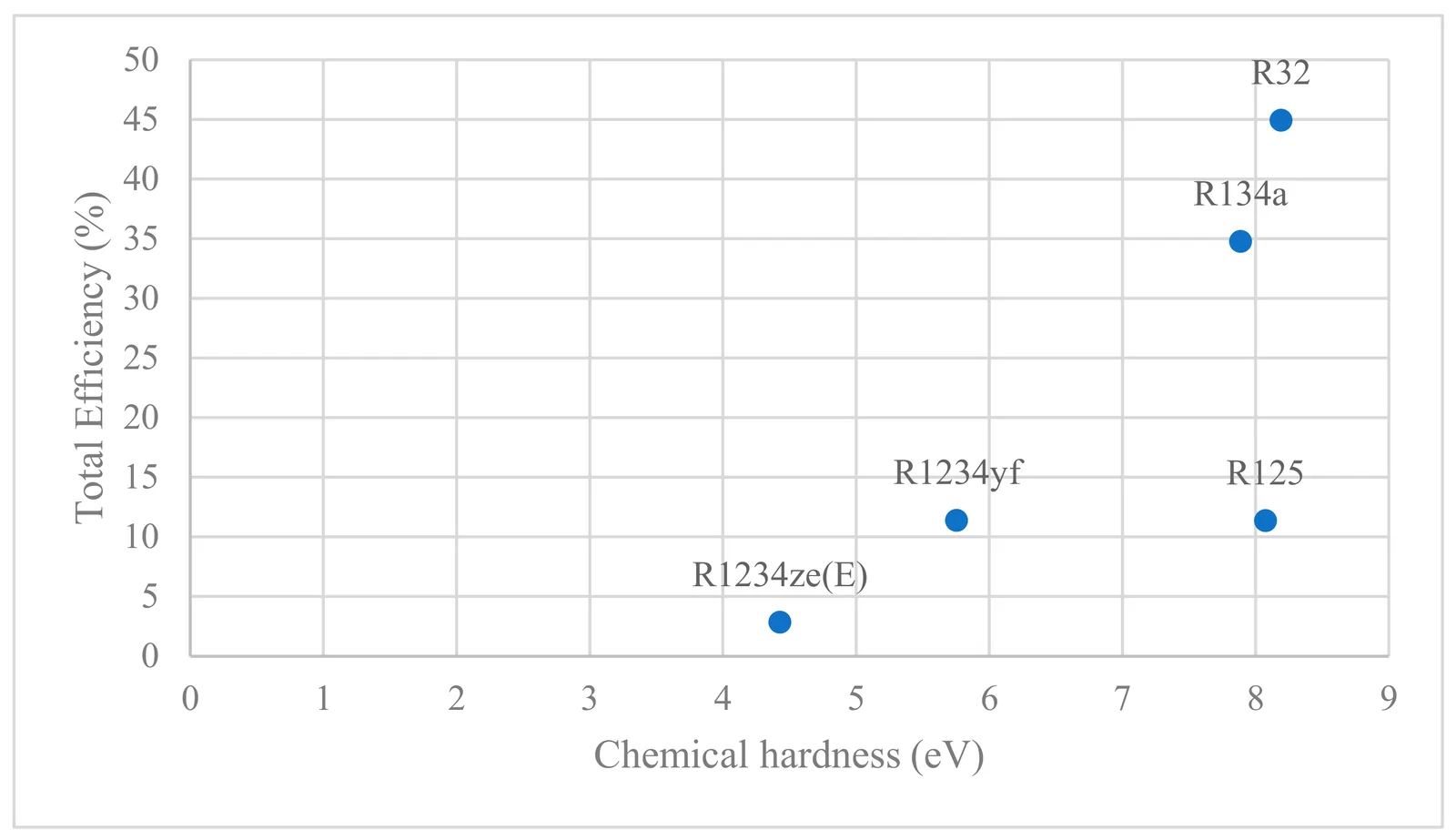
Chemical hardness and stability analysis of R448A refrigerant components.

Figure 12 presents the relationship between chemical hardness, derived from the HOMO–LUMO energy gap within the framework of Pearson’s hard–soft acid–base (HSAB) concept, and the total efficiency of the investigated refrigerant components. An apparent positive association is observed under the selected operating conditions within the limited set of refrigerants considered. R32, which has the highest chemical hardness value among the studied components, also exhibits the highest total efficiency value, whereas R1234yf and R1234ze(E) show lower chemical hardness values and lower total efficiency. This observed association should be interpreted descriptively and does not establish a causal or predictive relationship between chemical hardness and system-level performance. Chemical hardness is therefore considered a complementary molecular descriptor rather than a standalone indicator of refrigerant performance. The macroscopic efficiency of the system is governed by the thermophysical properties of the refrigerants, compressor work, cooling effect, and component-level exergy destruction.

### 2.5. Investigation of Electronic Properties

The highest occupied molecular orbital energy (E_HOMO_) and the lowest unoccupied molecular orbital energy (E_LUMO_) are fundamental electronic parameters that determine the chemical reactivity of molecules. The HOMO energy represents a molecule’s tendency to donate electrons (π-donor), while the LUMO energy reflects its ability to accept electrons (π-acceptor) [21]. In this study, the electronic structure parameters of the components of the R448A refrigerant (R32, R125, R134a, R1234yf, and R1234ze(E)) were calculated using the DFT/ωB97X-D functional and the DGDZVP2 basis set. The obtained E_HOMO_, E_LUMO_, and HOMO–LUMO energy gap (ΔE) values were evaluated comparatively.

Additionally, the ionization energy (I) and electron affinity (A) are key quantities describing the electronic stability and reactivity of molecules. Within the Koopmans approximation, the ionization energy was calculated approximately as I = −E_HOMO_, and the electron affinity as A = −E_LUMO_. Using these parameters, global reactivity indices such as electronegativity (χ), chemical hardness (η), and chemical softness (S) were derived [22], enabling a quantitative assessment of the molecules’ tendency for electron transfer.

In this context, all electronic structure and global reactivity parameters, including E_HOMO_, E_LUMO_, the energy gap between HOMO and LUMO (ΔE), ionization energy (I), electron affinity (A), electronegativity (χ), chemical hardness (η), and chemical softness (S), were calculated for all components of the R448A refrigerant. These calculations provide insight into the relative electronic stability and reactivity of each molecule [23]. The comparative results are presented in Table 5, offering a clear overview of how the molecular electronic properties of R32, R125, R134a, R1234yf, and R1234ze(E) contribute to the overall thermodynamic and chemical behavior of the R448A mixture.

The calculated electronic structure parameters in Table 5 provide valuable insight into the relative chemical stability and reactivity of the R448A refrigerant components. Molecules with lower HOMO–LUMO energy gaps (ΔE) and smaller chemical hardness (η) generally exhibit higher chemical softness, indicating greater reactivity. According to the results, R134a exhibits a lower HOMO energy (−12.60 eV) compared to R1234yf (−10.56 eV), suggesting that R134a has a lower tendency to donate electrons. Similarly, the LUMO energy of R134a (3.17 eV) is higher than that of R1234yf (0.95 eV), indicating that R1234yf is more prone to accept electrons. The HOMO–LUMO gap further confirms this trend, with R134a having a larger gap (15.78 eV) than R1234yf (11.51 eV), highlighting the higher stability of R134a and the greater chemical reactivity of R1234yf.

Ionization energies (I) and electron affinities (A) follow the same trend, with R134a being more resistant to losing electrons. Global reactivity descriptors, including chemical potential [24] (χ), chemical hardness (η), and softness (S), indicate that R1234yf is softer and therefore more susceptible to chemical reactions. Total electronic energies (E_TOTAL_) corroborate these observations, confirming the relative stability of the components. In combination, these density functional theory (DFT) results allow for a quantitative comparison of the electronic properties and reactivity trends of all five R448A components, providing a deeper understanding of how molecular structure influences thermodynamic behavior and performance in refrigeration applications.

**Figure 13:**
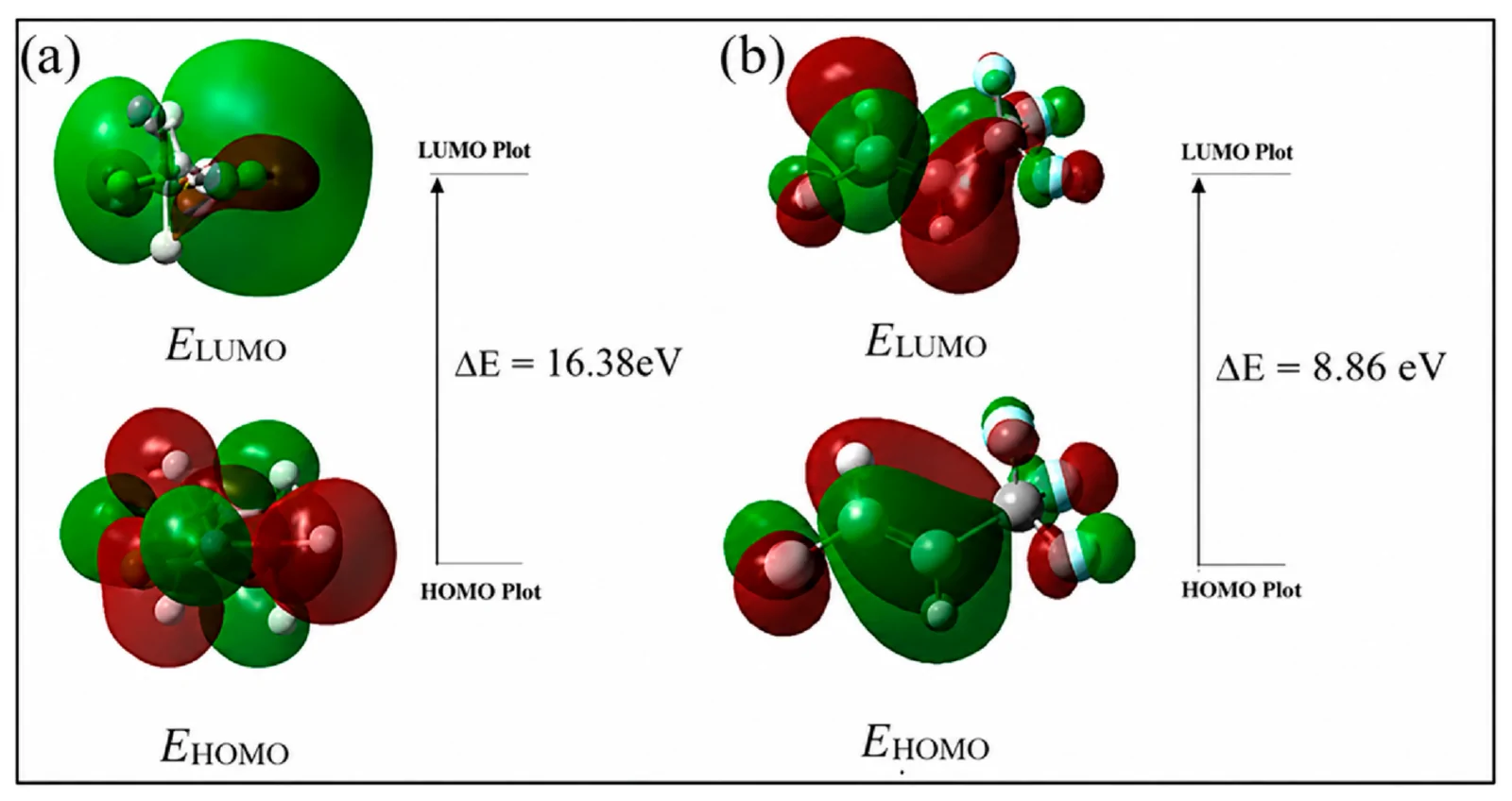
HOMO and LUMO isosurfaces of (**a**) R32 and (**b**) R1234ze(E) calculated at the DFT/ωB97X-D/DGDZVP2 level.

Significant differences in electronic structure and chemical reactivity exist among the components constituting the R448A refrigerant. Therefore, in the visual presentation of the HOMO–LUMO analyses, two characteristic components exhibiting extreme behaviors representative of the mixture were selected. In this context, R32, which exhibits the widest HOMO–LUMO energy gap and high electronic stability, and R1234ze(E), which shows the narrowest energy gap and high chemical reactivity, were evaluated together, and the corresponding HOMO and LUMO isosurfaces of these components are presented in Figure 13.

The HOMO and LUMO isosurfaces shown in Figure 13 reveal distinct electronic characteristics for the R32 and R1234ze(E) molecules. In both cases, the red and green regions represent opposite phases of the molecular orbitals. For R32, the HOMO is mainly localized around the carbon atom and along the C–F bonds, indicating a relatively stable electron-donating [25] behavior. In contrast, for R1234ze(E), the HOMO and LUMO are predominantly distributed over the C=C double-bond regions, reflecting enhanced electron delocalization and higher chemical reactivity [26].

## 3. Computational Studies

In this study, the molecular-level behavior of the R448A refrigerant and its individual components (R32, R125, R134a, R1234yf, and R1234ze(E)) was investigated using quantum chemical methods. All calculations were performed using the Gaussian 09 software (Revision D.01) [27] within the framework of Density Functional Theory (DFT) [28], employing the ωB97X-D [29] hybrid functional in conjunction with the DGDZVP2 [30] basis set. The ωB97X-D functional was selected due to its reliable description of long-range electronic interactions and dispersion effects, which are particularly important for halogenated molecules, while the DGDZVP2 basis set provides a balanced treatment of valence and polarization effects. Frequency calculations were performed after geometry optimization, and no imaginary frequencies were obtained, confirming that all optimized structures correspond to stable minima on the potential energy surface.

Since R448A is a zeotropic mixture, quantum chemical calculations were performed separately for its individual components (R32, R125, R134a, R1234yf, and R1234ze(E)) to obtain intrinsic molecular descriptors. Mixture-level effects, including component interactions, temperature glide, and non-ideal mixing behavior, were considered within the thermodynamic cycle analysis rather than isolated molecular calculations. This approach enables the determination of intrinsic molecular descriptors that can be used to interpret macroscopic thermodynamic and exergetic performance trends. It should be noted that the molecular structures considered in this study represent isolated gas-phase species, and no crystalline-phase effects were included in the quantum chemical calculations. Therefore, the calculated molecular descriptors are considered complementary molecular-level indicators for interpreting system-level performance trends rather than direct substitutes for bulk thermodynamic properties.

Geometry optimizations were carried out for all molecules. From these calculations, the internal energy (U), enthalpy (H), Gibbs free energy (G), and heat capacity at constant volume (Cv) were obtained.

Based on the optimized geometries, electronic properties including frontier molecular orbital energy levels (HOMO–LUMO), ionization potential, electron affinity, electronegativity, chemical hardness, and softness were calculated. These global reactivity descriptors were employed to quantitatively assess the chemical stability and reactivity of each component. The obtained quantum chemical and thermodynamic parameters were subsequently correlated with the macroscopic exergy analysis results derived from Rankine–VCR system simulations.

## 4. Conclusions

In this study, the performance of different generations of refrigerants used in marine waste heat recovery systems was analyzed by integrating thermodynamic and quantum chemical perspectives. Based on the obtained theoretical and simulation-based results, the following key conclusions can be drawn:

Within the limited set of refrigerants investigated, an apparent association was observed between chemical hardness and Second Law efficiency. R32 exhibited the highest chemical hardness value (8.19 eV) and the highest exergy efficiency, whereas the HFO refrigerants R1234ze(E) and R1234yf exhibited lower chemical hardness values and comparatively lower exergy efficiencies. However, this observed relationship is descriptive and does not establish a causal or predictive relationship between chemical hardness and system-level performance. The DFT calculations provide complementary information on the electronic structure and reactivity characteristics of the investigated refrigerant molecules.

The calculated standard molar entropy values were higher for the more structurally complex refrigerant components, such as R125 and the HFO refrigerants, within the investigated set. R32 exhibited a comparatively lower entropy value (58.9 cal/mol K). These results describe differences in the molecular thermodynamic characteristics of the investigated refrigerants; however, the observed differences in entropy should not be interpreted as direct evidence of a causal relationship with entropy generation or internal irreversibilities at the system level. The DFT-derived vibrational analysis provides complementary information on the molecular degrees of freedom associated with these differences.

Molecular heat capacity (Cv) showed differences among the investigated refrigerants under the selected conditions. These differences provide complementary information on the molecular thermal characteristics of the refrigerants. However, the observed variation in Cv should be interpreted as a descriptive molecular-level association and not as evidence of a direct effect on compressor load or system-level coefficient of performance (COP). The DFT-based vibrational and rotational analyses provide complementary information on the molecular characteristics associated with these variations.

Quantum corrections and thermal enthalpy capacities were also analyzed. Under the selected operating conditions, lower quantum mechanical “Thermal Enthalpy Corrections” were observed for refrigerants exhibiting favorable thermodynamic characteristics. R32 exhibited the highest cooling load per unit mass (~291.5 kJ/kg) among the investigated refrigerants. These results, supported by DFT-computed zero-point and thermal energies, provide complementary molecular-level information for interpreting the calculated thermodynamic results. However, these quantum-chemical descriptors should be regarded as descriptive indicators rather than predictive determinants of refrigerant performance.

The zeotropic mixture R448A exhibited lower thermodynamic performance than pure R32 but higher performance than R134a and R125 under the investigated operating conditions. Within the set of refrigerants considered, R448A therefore represents a comparatively favorable alternative. The molecular and thermodynamic characteristics of its constituent components provide complementary information for interpreting the observed performance differences; however, these characteristics should not be regarded as direct causal determinants of system-level performance.

Finally, design and cost considerations were evaluated in relation to molecular weight, energy density, and the required mass flow rate. R32 achieved the lowest required mass flow rate (0.068 kg/s) under the investigated conditions, primarily due to its favorable thermodynamic properties, particularly the higher enthalpy difference across the evaporator. Although its low molecular weight may contribute to certain transport-related characteristics, the mass flow requirement is fundamentally determined by the cycle enthalpy balance. The DFT results provide complementary molecular-level information, while the mass flow requirement is determined by the thermodynamic cycle and enthalpy balance under the investigated operating conditions.

In light of these results, future studies on refrigerant selection and system design should jointly consider fundamental thermodynamic performance criteria such as exergy efficiency, entropy generation, and coefficient of performance (COP), together with electronic structure parameters derived from density functional theory (DFT). This integrated approach may provide complementary information for the comparative assessment of refrigerants in marine waste heat recovery systems and other energy systems operating under high temperature gradients. Further studies involving larger refrigerant datasets and appropriate statistical and mechanistic analyses are required to determine whether the observed molecular-level associations can be generalized beyond the present set of refrigerants.

## Figures and Tables

**Table 1:** Design parameters and boundary conditions used in the thermodynamic analysis [11,12,13].

Component	Value	Component	Value
Turbine pressure ratio (P_4_/P_1_)	375	Rankine Condenser pressure	8 kPa
Turbine isentropic efficiency	0.85	VCR evaporator pressure	450 kPa
Pump isentropic efficiency	0.80	Dead-state temperature	25 °C
Shaft-coupling efficiency	0.96	Dead-state pressure	101.325 kPa
Compressor isentropic efficiency	0.90		

**Table 2:** Mass, energy, entropy, and exergy balance equations for the Rankine–VCR system components [14,15,16].

Component	Process	Mass Equation	Energy Balance	Entropy Balance	Exergy Balance and Efficiency
Pump	2-3	${\dot{m}}_{2}={\dot{m}}_{3}$	${\dot{W}}_{p}={\dot{m}}_{R}(h_{3}-h_{2})$	${\dot{m}}_{R}s_{2}+{\dot{s}}_{Gen}={\dot{m}}_{R}s_{3}$	${\dot{E}}_{X2}+{\dot{W}}_{P}={\dot{E}}_{x3}+{\dot{E}}_{XD,P}$ $\eta_{EX,P}=\left({\dot{E}}_{x3}-{\dot{E}}_{x2}\right)∕{\dot{W}}_{p}$
Rankine evaporator heated by exhaust gas	3-4	${\dot{m}}_{3}={\dot{m}}_{4}$	${\dot{Q}}_{gas}={\dot{m}}_{R}(h_{4}-h_{3})$	${\dot{m}}_{R}s_{3}+\frac{{\dot{Q}}_{gas}}{T_{source}}+{\dot{s}}_{Gen,EGin}={\dot{m}}_{R}s_{4}$	${\dot{E}}_{X3}+{\dot{E}}_{X,EGAS}={\dot{E}}_{x4}+{\dot{E}}_{XD,EGin}$ $\eta_{EX,EG}=\left({\dot{E}}_{x4}-{\dot{E}}_{x3}\right)∕\left({\dot{E}}_{Gin}-{\dot{E}}_{Gout}\right)$
Steam Turbine	4-1	${\dot{m}}_{4}={\dot{m}}_{1}$	${\dot{W}}_{ST}={\dot{m}}_{R}(h_{4}-h_{1})$	${\dot{m}}_{R}s_{4}+{\dot{s}}_{GenST}={\dot{m}}_{R}s_{1}$	${\dot{E}}_{X4}={\dot{E}}_{x1}+{\dot{W}}_{ST}+{\dot{E}}_{X,DST}$ $\eta_{EX,ST}={\dot{W}}_{ST}/\left({\dot{E}}_{x4}-{\dot{E}}_{x1}\right)$
Condenser (Rankine)	1-2	${\dot{m}}_{1}={\dot{m}}_{2}$	${\dot{Q}}_{CD,ran}={\dot{m}}_{R}(h_{1}-h_{2})$	${\dot{m}}_{R}s_{2}+\frac{{\dot{Q}}_{CD,R}}{T_{0}}={\dot{m}}_{R}s_{1}+{\dot{s}}_{Gen,CD,R}$	${\dot{E}}_{X1}={\dot{E}}_{x2}+{\dot{E}}_{XQ,CD,RAN}+{\dot{E}}_{XD,CD,RAN}$
Compressor	8-5	${\dot{m}}_{5}={\dot{m}}_{8}$	${\dot{W}}_{Comp}={\dot{m}}_{ref}(h_{5}-h_{8})$	${\dot{m}}_{ref}s_{8}+{\dot{s}}_{Gen,Comp}={\dot{m}}_{ref}s_{5}$	${\dot{E}}_{X8}+{\dot{W}}_{Comp}={\dot{E}}_{X5}+{\dot{E}}_{XD,Comp}$ $\eta_{EX,Comp}=\left({\dot{E}}_{X5}-{\dot{E}}_{X8}\right)∕{\dot{W}}_{Comp}$
Condenser (VCR)	5-6	${\dot{m}}_{5}={\dot{m}}_{6}$	${\dot{Q}}_{CD,VCR}={\dot{m}}_{ref}(h_{5}-h_{6})$	${\dot{m}}_{ref}s_{6}+\frac{{\dot{Q}}_{CD,VCR}}{T_{0}}={\dot{m}}_{ref}s_{5}+{\dot{s}}_{Gen,CD,VCR}$	${\dot{E}}_{X5}={\dot{E}}_{x6}+{\dot{E}}_{XQ,CD,vcr}+{\dot{E}}_{XD,CD,VCR}$
Expansion Valve	6-7	${\dot{m}}_{6}={\dot{m}}_{7}$	$h_{6}=h_{7}$	${\dot{m}}_{ref}s_{6}+{\dot{s}}_{Gen,Valve}={\dot{m}}_{ref}s_{7}$	${\dot{E}}_{X6}={\dot{E}}_{x7}+{\dot{E}}_{XD,Valve}$
Evaporator (VCR)	7-8	${\dot{m}}_{7}={\dot{m}}_{8}$	${\dot{Q}}_{Evap,VCR}={\dot{m}}_{ref}(h_{8}-h_{7})$	${\dot{m}}_{ref}s_{7}+\frac{{\dot{Q}}_{Evap,VCR}}{T_{Room}}+{\dot{s}}_{Gen,EvapVCR}={\dot{m}}_{ref}s_{8}$	${\dot{E}}_{X7}+{\dot{E}}_{XQ,VCR}={\dot{E}}_{x8}+{\dot{E}}_{XD,VCR}$
**COP**		**Fluid Mass Flow Rate**		**Total Entropy Generation**	**Total Exergy Efficiency**
$COP=\frac{{\dot{Q}}_{L}}{{\dot{W}}_{Comp}}=\frac{h_{8}-h_{7}}{h_{5}-h_{8}}$		${\dot{m}}_{ref}=\frac{{\dot{Q}}_{L}}{h_{8}-h_{7}}$		${\dot{s}}_{Gen,Total}=\sum {\dot{S}}_{Gen,Materials}$	$\eta_{e}=\frac{{\dot{E}}_{XQ,Cool}}{{\dot{E}}_{XGAS,IN}-{\dot{E}}_{XGAS,OUT}}$

**Table 3:** Experimental and optimized geometric parameters of the R448A refrigerant components calculated at the DFT/ωB97X-D/DGDZVP2 level.

R32			R125			R134a			R1234yf			R1234ze		
**Bond Length (Å)**	**Exp.**	**Opt.**	**Bond Length (Å)**	**Exp.**	**Opt.**	**Bond Length (Å)**	**Exp.**	**Opt.**	**Bond Length (Å)**	**Exp.**	**Opt.**	**Bond Length (Å)**	**Exp.**	**Opt.**
F1–C3	1.35	1.36	F1–C6	1.35	1.34	F4–C1	1.32	1.34	F1–C6	1.33	1.34	F1–C4	1.34	1.34
F2–C3	1.35	1.36	F3–C6	1.35	1.33	F3–C1	1.32	1.34	F2–C6	1.35	1.34	C4–C5	1.32	1.33
			F2–C6	1.35	1.34	F5–C1	1.32	1.35	F4–C6	1.34	1.34	C5–C6	1.48	1.50
			C6–C7	1.50	1.55	C1–C2	1.52	1.53	C6–C5	1.49	1.52	C6–F2	1.33	1.35
			F4–C7	1.36	1.35	C2–F5	1.35	1.38	C5–C7	1.30	1.32	C6–F3	1.35	1.35
			F5–C7	1.36	1.35				F3–C5	1.34	1.34	C6–F9	1.35	1.35
**Bond** **Angles (^o^)**	**Exp.**	**Opt.**	**Bond ** ** Angles (^o^)**	**Exp.**	**Opt.**	**Bond ** ** Angles (^o^)**	**Exp.**	**Opt.**	**Bond ** ** Angles (^o^)**	**Exp.**	**Opt.**	**Bond ** ** Angles (^o^)**	**Exp.**	**Opt.**
F1–C3–F2	107.09	108.74	F1–C6–F2	107.78	108.49	F3–C1-F4	107.34	107.97	F1–C6–F2	107.64	108.12	F1–C4–C5	120.00	121.30
			F1–C6–F3	108.02	108.96	F3–C1-F5	107.71	108.12	F1–C6–F4	107.90	108.12	C4–C5–C6	121.52	121.29
			F2–C6–F3	108.02	108.96	F3–C1–C2	109.14	111.94	F1–C6–C5	112.04	111.01	C5–C6–F2	113.17	112.73
			F1–C6–C7	110.88	109.33	F5–C1–F4	108.06	108.12	F2–C6–F4	105.92	107.47	C5–C6–F3	111.81	111.19
			F2–C6–C7	110.88	109.32	F5–C1–C2	110.68	108.61	F2–C6–C5	111.32	110.99	C5–C6–F9	111.81	111.19
			F4–C7–F5	106.93	109.15	F4–C1–C2	111.24	111.94	C6–C5–F3	109.94	111.08	F2–C6–F3	106.98	107.36
						C1–C2–F6	109.14	109.88	C6–C5–C7	126.47	125.84	F2–C6–F9	106.98	107.36
									F3–C5–C7	123.59	123.08	F3–C6–F9	105.62	106.71

**Table 4:** Thermodynamic parameters of the R448A refrigerant components calculated at the DFT/ωB97X-D/DGDZVP2 level.

	R32	R125	R134a	R1234yf	R1234ze(E)
Thermal energy, E (Kcal/mol)					
Rotational	0.889	0.889	0.889	0.889	0.889
Translational	0.889	0.889	0.889	0.889	0.889
Vibrational	20.948	25.938	30.443	33.532	33.838
Total	22.725	27.715	32.220	35.310	35.616
Heat capacity, C_v_ (cal/mol K)					
Rotational	2.981	2.981	2.981	2.981	2.981
Translational	2.981	2.981	2.981	2.981	2.981
Vibrational	2.250	14.388	2.981	15.803	15.611
Total	8.212	20.350	18.028	21.765	21.572
Entropy, S (cal/mol K)					
Rotational	20.375	27.328	26.495	27.292	27.779
Translational	37.769	40.261	39.777	40.109	40.109
Vibrational	0.814	12.030	9.324	12.803	13.028
Total	58.958	79.620	75.596	80.203	80.916
Rotational constants (GHz)					
A	49.27297	3.64458	5.28760	3.67945	5.29386
B	10.43788	2.38416	2.76165	2.44261	1.44821
C	9.13689	1.97723	2.72048	1.98255	1.42285
Rotational temperature (K)					
A	2.36473	0.17491	0.25376	0.17659	0.25407
B	0.50094	0.11442	0.13254	0.11723	0.06950
C	0.43850	0.09489	0.13056	0.09515	0.06829
Thermal properties (Hartree/particle)					
Zero-point correction	0.033089	0.038068	0.045889	0.049983	0.050392
Thermal correction to Energy	0.036215	0.044167	0.051346	0.056270	0.056757
Thermal correction to Enthalpy	0.037159	0.045111	0.052290	0.057214	0.057702
Thermal correction to Gibbs Free Energy	0.009146	0.007282	0.016372	0.019107	0.019256
Sum of electronic and zero-point Energies	−238.963953	−576.013609	−476.762118	−514.826297	−514.829703
Sum of electronic and thermal Energies	−238.960828	−576.007509	−476.756661	−514.820010	−514.823338
Sum of electronic and thermal Enthalpies	−238.959884	−576.006565	−476.755717	−514.819066	−514.822393
Sum of electronic and thermal Free Energies	−238.987897	−576.044395	−476.791635	−514.857173	−514.860839
Zero point vibrational energy (kcal/mol)	20.76390	23.88778	28.79592	31.36462	31.62167

**Table 5:** Theoretically calculated electronic structure parameters of the R448A refrigerant components calculated at the DFT/ωB97X-D/DGDZVP2 level.

	R32	R125	R134a	R1234yf	R1234ze(E)
E_HOMO_ (eV)	−12.0068	−12.6756	−12.6035	−10.5629	−7.8799
E_LUMO_ (eV)	4.3783	3.4735	3.1726	0.9489	0.9772
ΔE = E_LUMO_ − E_HOMO_ (eV)	16.3851	16.1492	15.7761	11.5118	8.8570
I (eV)	12.0068	12.6756	12.6035	10.5629	7.8799
A (eV)	−4.3783	−3.4735	−3.1726	−0.9489	−0.9772
χ (eV)	3.8142	4.6010	4.7155	4.8070	3.4514
η (eV)	8.1925	8.0746	7.8880	5.7559	4.4285
S (eV^−1^)	0.0610	0.0619	0.0634	0.0869	0.1129
E_TOTAL_ (Hartree)	−238.99704	−576.05168	−476.80801	−514.87628	−514.8801

## Data Availability

The original contributions presented in this study are included in the article. Further inquiries can be directed to the corresponding author.

## Abbreviations

R448A: Non-flammable zeotropic refrigerant blend composed of the following components: R32: Difluoromethane; R125: Pentafluoroethane; R134a: 1,1,1,2-Tetrafluoroethane; R1234yf: 2,3,3,3-Tetrafluoropropene; R1234ze(E): trans-1,3,3,3-Tetrafluoropropene; COP: Coefficient of Performance; DFT: Density Functional Theory; ωB97X-D: Range-separated hybrid density functional with empirical dispersion correction; DGDZVP2: A double-zeta valence basis set including polarization functions, widely used in density functional theory (DFT) calculations for improved accuracy in molecular property predictions. ED: Exergy Destruction; η: Exergy Efficiency; W: Compressor Work; $\dot{Q}\mathrm{cond}$: Heat released from the condenser; $\dot{Q}\mathrm{cool}$: Cooling capacity; GWP: Global Warming Potential; HFO: Hydrofluoroolefin; HFC: Hydrofluorocarbon; CFC: Chlorofluorocarbon; HCFC: Hydrochlorofluorocarbon.
